# Supplementary material for: Trend and determinants of unmet need for family planning among married women in Ethiopia, evidence from Ethiopian demographic and health survey 2000–2016; multilevel analysis
Source: PLoS One. 2024 Jan 2;19(1):e0296382. doi: 10.1371/journal.pone.0296382 (PMC10760879; doi:10.1371/journal.pone.0296382)
Supplement: Attachment — Submitted filename: Response to reviewer.docx [file pone.0296382.s002.docx]

**Here are the responses for points raised**

1. Contribution of the study is not clear in the introduction section. Just saying that previous studies have not considered the clustering effects is not enough to claim the academic contribution of the study. What's the scholarly contribution of this study?

**Response:** we have added in the revised manuscript.

2. Definitions of all variables should be given in a table. There are few undefined variables e.g., "parity" is not defined and another ambigious variable is "reason for not using".

**Response: we have fixed it now.**

3. Wealth index is constructed by PCA and some discrete variables are used in this exercise, my question is regarding the use of correlation measure in PCA, is this the buil-in Pearson correlation measure or authors have used tetrachoric correlation? If Pearson is used then results are not valid for wealth index (see Islam, T. U., & Rizwan, M. (2020). Comparison of correlation measures for nominal data. Communications in Statistics-Simulation and Computation, 51(3), 698-714).

**Response:** Thank you for recommending the article and we referred it. for the construction of wealth index, variables were taken from Household Recode file. Discrete variables used for computing wealth index after recoded to a dichotomous variable/dummy variable based on the DHS standard criteria first and coded to 0/1. Then, biseral correlation (one of tetrachoric correlation) was used.

4. Multilevel modelling: Give detailed econometric methodology with underlying distribution assumptions (if any). Also, provide the diagnostic statistics to validate the model. The model is constructed after checking the random effects

**Response: we have added a detail about the multilevel model in the revised manuscript. Regarding econometric modelling, we do not have that.** I could not find any discussion on the explained variation at the individual and community level.

**Response:** In the empty model, we simply aim to identify a possible contextual phenomenon that can be quantified by clustering of unmet need within neighborhoods. The ICC (intra-cluster correlation coefficient), MOR (median odds ratio), PCV(proportion change in variance) an the significant chi square test indicates the clustering effect in unmet need. We used these results to check the whether multilevel model is required or not. However, we added in the discussion in the revised manuscript.

5. Model 3 is unexplained.

**Response:** Model 3 is adjusted for only community level variables (as mentioned in the manuscript). However, the best-fitted model based on model selection criteria (AIC and BIC), model 4 is best fitted which was reported as final model.

**Reviewer #2:** I suggest that unmet need according to different life-stages should be explored as well, particularly the period of one year postpartum

1. Thank you for your suggestion. We addressed in revised manuscript.
